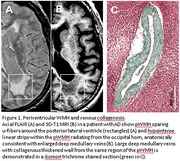# Periventricular white matter hyperintensities co‐localize with deep intramedullary venules, sparing compact fiber tracts and can increase or decrease over time in Alzheimer's disease

**DOI:** 10.1002/alz70856_099476

**Published:** 2025-12-25

**Authors:** Fuqiang Gao, Joel Ramirez, Melissa F Holmes, Julia Keith, Mario Masellis, Richard H. Swartz, Sandra E. Black

**Affiliations:** ^1^ Dr. Sandra Black Centre for Brain Resilience and Recovery, Sunnybrook Research Institute, Toronto, ON, Canada; ^2^ Dr. Sandra E. Black Centre for Brain Resilience and Recovery, LC Campbell Cognitive Neurology, Hurvitz Brain Sciences Program, Sunnybrook Research Institute, University of Toronto, Toronto, ON, Canada; ^3^ University of Toronto Scarborough, Toronto, ON, Canada; ^4^ Sunnybrook Health Sciences Centre, Toronto, ON, Canada; ^5^ Division of Neurology, Department of Medicine, Sunnybrook Health Sciences Centre, Toronto, ON, Canada; ^6^ Sunnybrook Research Institute, Toronto, ON, Canada; ^7^ Cognitive and Movement Disorders Clinic, Sunnybrook Health Sciences Center, Toronto, ON, Canada; ^8^ University of Toronto, Toronto, ON, Canada; ^9^ Hurvitz Brain Sciences Program, Toronto, ON, Canada; ^10^ Sunnybrook Health Sciences Centre, University of Toronto, Toronto, ON, Canada

## Abstract

**Background:**

Periventricular white matter hyperintensities (pWMH), commonly thought to be ischemic and demyelinating in origin, are prevalent in Alzheimer's disease (AD). However, occlusive collagenosis of deep medullary veins (DMVs), a venulopathy has been implicated in pWMH. Not only leakage, but also perivascular lymphatic system stasis may lead to chronic excessive extracellular fluid accumulation. In vivo, we investigated collocation of confluent pWMH with DMVs and radiological evidence of pWMH edema in AD, in conjunction with pathological correlation.

**Method:**

88 AD and 33 controls (age=76) with confluent pWMH on T2/FLAIR were included. DMVs were defined as linear streaks on T1/inverted‐T2 radiating from the subependymal region of the lateral ventricle. Spatial relationships of confluent pWMH was measured by counting the number of DMVs in each confluent pWMH. Radiological signs of edema were determined by whether compact white matter tracts (including optic radiation, fronto‐occipital fasciculus, thalamofrontal tract and u‐fibers) were spared within or around pWMH. Also pWMH changes over time were analyzed, assuming WMH would exhibit dynamic changes if they are edema related. Perivascular spaces and lacunes were also quantified. Thirteen imaging‐pathological correlations were available to validate imaging findings.

**Result:**

DMVs were identified in 283 discrete confluent pWMH across all participants. Significant associations were demonstrated between pWMH volume and total number of DMVs depicted within pWMH. Co‐location of pWMH with DMVs appeared to be unique by comparing them to regions without pWM. Compact fiber tracts were spared by confluent pWMH in 95% overall, and 24% of pWMH volumes decreased over time. Pathologically, using trichrome staining, venous collagenosis (wall thickening, stenosis or occlusion) of large (*p* = 0.012) and smaller venules (*p* = 0.036) were significant predictors of pWMH. Demyelination using luxol blue contributed but did not survive multiple regression.

**Conclusion:**

Confluent pWMH clearly mapped to visualizable DMVs, suggesting venous insufficiency due to collagenosis. Compact fiber tract sparing and reversible progression of pWMH provided in vivo evidence of chronic edema. Our findings support the notion that venous insufficiency of DMVs is the main substrate of pWMH, reflecting vasogenic edema induced from increased venous pressure and extracellular fluid accumulation from reduced interstitial fluid circulation along the glymphatic perivenous spaces.